# Neuro-Renal Crosstalk Across the Chronic Kidney Disease Spectrum: Stage-Dependent Molecular Mechanisms of Cognitive Impairment—An Integrative Review

**DOI:** 10.3390/ijms27156550

**Published:** 2026-07-23

**Authors:** Piotr Olejnik, Dominika Kurzawa, Jolanta Małyszko, Aleksandra Golenia

**Affiliations:** 1 Department of Neurology, Medical University of Warsaw, 02-097 Warsaw, Poland; 2 Doctoral School, Medical University of Warsaw, 02-093 Warsaw, Poland; 3 Department of Neurology, University Clinical Center, 02-097 Warsaw, Poland; dominika.kurzawa@uckwum.pl; 4 Department of Nephrology, Dialysis and Internal Medicine, Medical University of Warsaw, 02-097 Warsaw, Poland; jolmal@poczta.onet.pl

**Keywords:** chronic kidney disease, kidney failure, cognitive impairment, dementia, cerebral small vessel disease, molecular mechanisms, uremic toxins

## Abstract

Chronic kidney disease (CKD) is a major public health problem worldwide, affecting over 10% of the global population, and its prevalence continues to increase. In addition to classic comorbidities, including cardiovascular and metabolic disorders, cognitive impairment (CI) has recently been recognized as a serious complication of CKD. CI can be observed across the entire spectrum of CKD, from G1 to G5 stages, independent of age-related changes. The kidney–brain axis seems to provide a conceptual framework in which microvascular disease, blood–brain barrier disruption, uremic toxins, oxidative stress, systemic inflammation, and consequent neuroinflammation converge to promote CI. This narrative review aims to synthesize the existing evidence on stage-dependent molecular mechanisms of CI in CKD, integrating clinical observations with proposed molecular and pathophysiological mechanisms. Because most studies investigating CI in CKD rely primarily on eGFR-based staging, our analysis was structured around GFR categories, assessing early-to-moderate CKD, defined according to KDIGO as G1–G3 stages, advanced CKD stages, including G4–G5 not yet requiring kidney replacement therapies, and kidney failure treated with different modalities of kidney replacement therapy, such as peritoneal dialysis, hemodialysis, and kidney transplantation. In addition to clinical observations and the burden of CI in CKD, we describe the potential stage-dependent pathomechanisms underlying vascular-executive, uremic-toxic, inflammatory, dialysis-hemodynamic, and post-transplant medication-related profiles as a continuum of cognitive disorders in individuals with CKD.

## 1. Introduction

Chronic kidney disease (CKD) is one of the major public health problems worldwide, affecting over 10% of the global population [1]. However, its prevalence is constantly rising, which is attributable to an increase in the prevalence of the most common causes of CKD, including diabetes mellitus, heart disease, hypertension, and aging [2]. In 2017, there were 697.5 million cases of CKD in all stages, whereas in 2023 this number reached 788 million, representing an increase of nearly 100 million cases in six years and more than 400 million cases compared to 1990 [3,4]. Additionally, in 2019, CKD was ranked 10th among the leading causes of death worldwide, but by 2023 it had risen to 9th place [4,5]. Ultimately, it is estimated that by 2040 CKD will rank 5th in this category, ahead of all types of cancer, Alzheimer’s disease, and diabetes [5]. Notably, high mortality is not limited to advanced CKD but gradually increases with disease progression, as CKD affects the risk of morbidity associated with other conditions, mainly cardiovascular disease [5].

Given its increasing prevalence, mortality burden, and systemic clinical consequences, a standardized definition and classification of CKD are essential for its diagnosis, risk stratification, and management. According to the 2024 update of the Kidney Disease: Improving Global Outcomes (KDIGO) Clinical Practice Guideline for the Evaluation and Management of Chronic Kidney Disease, CKD is defined as an abnormality of kidney structure or function that persists for more than 3 months and has implications for health. CKD is classified according to Cause, estimated Glomerular Filtration Rate (eGFR) category (G1–G5), and Albuminuria category (A1–A3), collectively referred to as the CGA (Cause/Glomerular filtration rate/Albuminuria) classification [6,7]. This classification is clinically relevant not only for estimating renal prognosis, but also for assessing the risk of systemic complications [7].

Beyond its classic comorbidities, such as hypertension, heart failure, and metabolic disorders, cognitive impairment (CI) is increasingly recognized as another serious complication of CKD [8,9,10]. CI can be broadly defined as a decline from an individual’s previous level of functioning in one or more cognitive domains, including complex attention, executive function, learning and memory, language, perceptual-motor function, and social cognition [11]. Harvey (2019) further emphasizes that cognitive functioning results from the interaction of multiple cognitive processes, indicating that CI may extend beyond deficits in specific domains alone [12]. As higher-order abilities, like reasoning and problem-solving, depend on the integration of several cognitive processes [12]. In the context of CKD, cognition is typically conceptualized as a multidimensional construct encompassing both global cognitive functioning and specific cognitive domains, with executive function, attention, and processing speed appearing to be particularly vulnerable among individuals with CKD [13,14].

The first meta-analysis to analyze the link between CKD and cognitive decline, conducted by Etgen et al. in 2012, showed that individuals with CKD had a significantly elevated risk of CI in comparison with those without CKD, in both cross-sectional (Odds Ratio [OR]: 1.65; 95% Confidence Interval: 1.32–2.05; *p* < 0.001) and longitudinal (OR 1.39; 95% Confidence Interval: 1.15–1.68; *p* < 0.001) study designs [15]. According to a systematic review conducted by Brodski and co-workers (2019), CI can be observed across the entire spectrum of CKD, from G1 to G5 CKD individuals, independent of age-related changes [16]. Although there is no standardized cognitive screen dedicated to CKD patients, and therefore studies are difficult to compare directly, the highest prevalence of CI is observed in hemodialysis (HD) individuals, followed by those treated with peritoneal dialysis (PD), patients not requiring kidney replacement therapy (KRT), and kidney transplant (KTx) recipients [17,18].

CI constitutes one of the most clinically consequential extrarenal manifestations of CKD, as it affects adherence to medical treatment, dialysis decision-making, frailty, hospitalization rate, and mortality risk, as well as increases healthcare costs [19]. It also affects transplant eligibility, as CI reduces patients’ likelihood of being listed for KTx [20].

The concept of kidney–brain axis seems to provide a conceptual framework in which microvascular disease, blood–brain barrier (BBB) disruption, uremic toxins, oxidative stress, systemic inflammation, and consequent neuroinflammation converge to promote cognitive decline [21]. Nonetheless, the exact mechanism leading to CI in individuals with CKD remains uncertain. Available data suggest that CI in CKD is likely multifactorial and may involve stage-dependent pathomechanisms (summarized in Table 1) [22]. Nonetheless, these mechanisms have not yet been fully integrated across the different stages of CKD and KRT modalities. This generates a gap in our knowledge, as the biological exposure profile of an individual with stage G1 CKD and albuminuria differs significantly from that of a person with stage G5 not requiring KRT affected with chronic uremia, a patient undergoing HD three times a week, or a KTx recipient treated with calcineurin inhibitors (CNI) [21].

Therefore, this narrative review aims to synthesize the existing evidence on stage-dependent molecular mechanisms of CI in CKD, integrating clinical observations with proposed pathophysiological pathways.

## 2. Methodology: Search Strategy

To provide a comprehensive and methodologically sound review, a literature search was conducted using the PubMed and Google Scholar databases, encompassing publications available until May 2026. The search strategy included a broad range of study types, including experimental and clinical studies, as well as review papers. The following keywords were used to identify relevant literature: ‘chronic kidney disease, ‘kidney failure’, ‘cognitive impairment’, ‘cognitive decline’, ‘pattern of affected domains’, and ‘molecular mechanisms’. Titles and abstracts were screened based on scientific relevance, and additional sources were identified through manual reference list checks to capture potentially overlooked articles. The inclusion criteria were limited to peer-reviewed full-text articles published in English. Non-peer-reviewed sources, such as preprints, and publications written in languages other than English were excluded to ensure the scientific quality and relevance of the studies included.

## 3. The Concept of Kidney–Brain Axis

The kidneys and the central nervous system (CNS) form a bidirectional regulatory network, commonly referred to as the kidney–brain axis. Under physiological conditions, brain–kidney communication contributes to systemic homeostasis primarily through autonomic and neuroendocrine pathways [23]. The sympathetic nervous system modulates the renal vascular tone, tubular sodium transport, renin secretion, and renal hemodynamics. Increased renal sympathetic nerve activity stimulates renin release, enhances tubular sodium reabsorption, reduces urinary sodium excretion, and, particularly during stronger sympathetic activation, may decrease the renal blood flow and GFR. Thus, CNS-mediated autonomic regulation can influence renal perfusion, filtration, and sodium handling [24].

**Figure 1:**
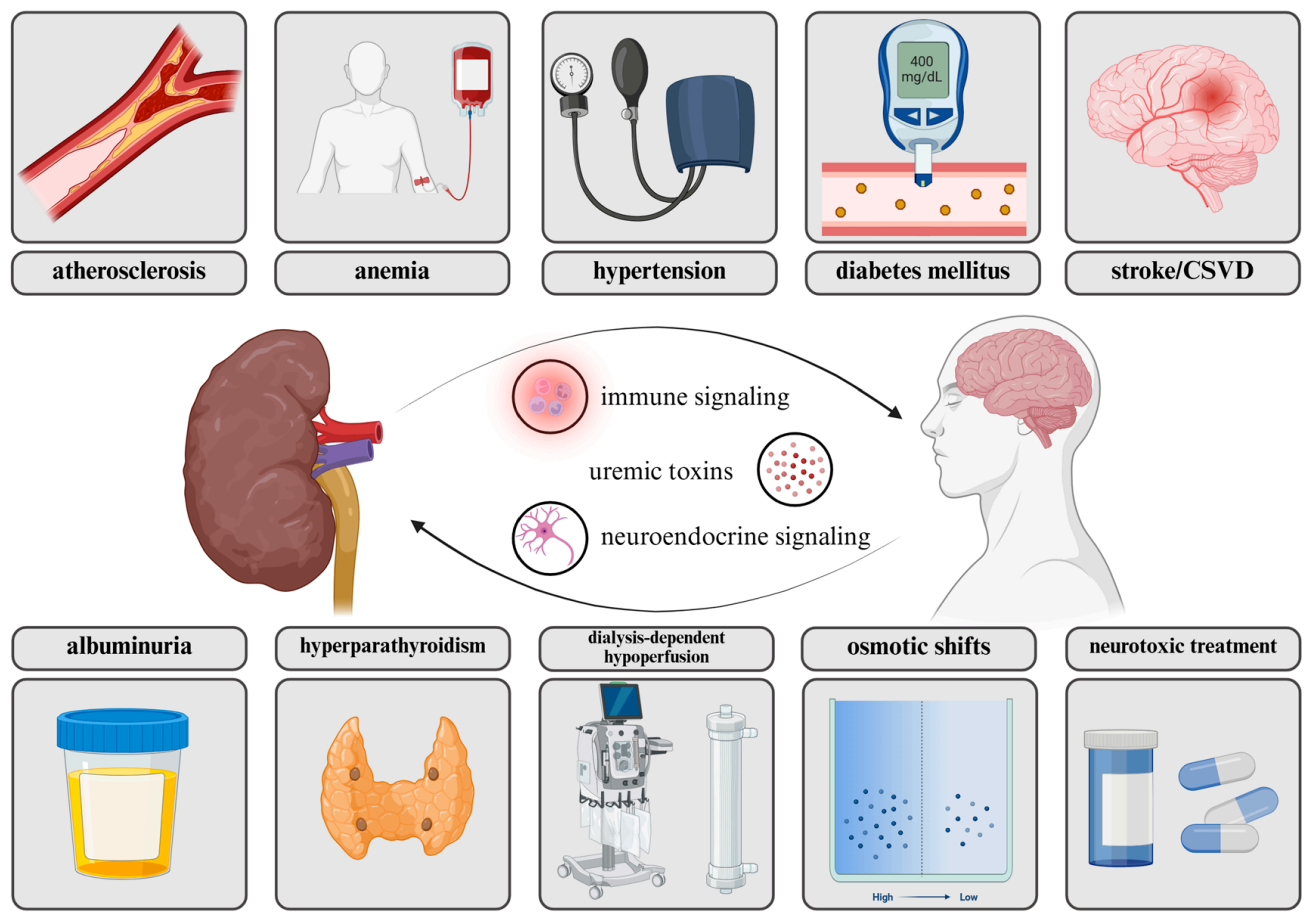
The kidney–brain axis and factors contributing to brain injury in the course of chronic kidney disease. The figure illustrates the bidirectional relationship between the kidney and the brain, mediated through immune signaling, uremic toxins, and neuroendocrine signaling. It highlights both classical vascular risk factors, including atherosclerosis, anemia, hypertension, diabetes mellitus, and stroke or cerebral small vessel disease (CSVD), as well as kidney disease-specific mechanisms. These include albuminuria, secondary hyperparathyroidism, dialysis-related hypoperfusion, osmotic shifts, and exposure to neurotoxic treatments. Together, these pathways may contribute to cerebrovascular injury, cognitive impairment, and other neurological complications in patients with kidney disease. Created in BioRender. Olejnik, P. (2026) https://BioRender.com/ym0y7ta.

In parallel, vasopressin, which is synthesized in the hypothalamus and released from the posterior pituitary gland, represents a key neuroendocrine pathway linking the brain and kidneys. By promoting water reabsorption in the distal nephron and collecting ducts, vasopressin primarily contributes to osmotic balance and extracellular fluid homeostasis and, therefore, participates in the regulation of blood pressure [25]. Conversely, kidney-derived signals, including uremic toxins, inflammatory mediators, vascular injury, and metabolic disturbances, may affect CNS function, particularly in patients with CKD. Therefore, disruption of this bidirectional kidney–brain communication may contribute to neurological complications, including CI [21]. Figure 1 illustrates the kidney–brain axis and the pathological conditions that may disrupt this relationship, incorporating both kidney disease-specific factors and classical vascular risk factors.

### 3.1. Shared Microvascular Vulnerability

The CNS and kidneys are both high-flow, low-resistance end organs that rely heavily on autoregulatory mechanisms. Their microvascular networks comprise small arteries, penetrating arterioles, capillaries, and venules, collectively referred to as the small vessels. Because of these shared hemodynamic and structural features, the brain and kidneys are similarly vulnerable to microvascular injury, which may evolve in parallel as part of a systemic small vessel disease (SVD) phenotype [26]. Accordingly, substantial research has focused on the association between CKD and cerebrovascular disorders [27]. For instance, a recent large multicenter prospective cohort study involving nearly 4000 patients with acute ischemic stroke from 13 centers showed that CKD was independently associated with a higher burden of cerebral SVD, including white matter hyperintensities (WMHs), lacunar infarctions, and cerebral microbleeds, even after adjustment for demographic, vascular, and stroke-related confounders [28].

Nevertheless, it remains uncertain whether cerebral SVD in CKD merely reflects shared comorbidities, including hypertension and diabetes mellitus, or whether it is also driven by some kidney-specific factors [27].

On the one hand, similarities in the histological architecture and functional organization of the cerebral and renal microvasculature make it plausible that traditional risk factors affect small vessels in both organs simultaneously, a concept referred to as the ‘strain vessel hypothesis’. According to this hypothesis, low-resistance small vessels are exposed to continuous high-flow perfusion, making them particularly vulnerable to hypertension-induced injuries [26]. But on the other hand, CKD-related disturbances in mineral metabolism, including phosphate retention, secondary hyperparathyroidism, loss of calcification inhibitors, and vascular smooth muscle cell transformation toward an osteogenic phenotype, may promote vascular calcification and arterial stiffness [27]. In addition, albuminuria may not only be interpreted as an indicator of renal dysfunction but also as a marker of systemic endothelial dysfunction, reflecting a vascular phenotype that can also manifest in the brain as SVD. Ultimately, proteinuria may contribute to dysregulated lipid metabolism through the urinary loss of lipoprotein-related proteins and altered lipoprotein transport, thereby promoting atherogenesis and further aggravating vascular injury [26].

### 3.2. Neurotoxicity of Uremic Toxins

As CKD progresses, uremic toxins gradually accumulate as a consequence of declining kidney function. More than 100 such compounds have been identified by the European Uremic Toxin Work Group and can be classified into three main groups: small water-soluble solutes, including urea and uric acid; protein-bound solutes, including indoxyl sulfate and p-cresyl sulfate; and middle molecules, such as β2-microglobulin [29]. Uremic toxicity is thought to be one of the potential causes of CI in individuals with CKD, participating in both main pathophysiological hypotheses: neurodegenerative and vascular [30,31]. Uremic toxins may contribute to CI in CKD through both vascular and direct cellular mechanisms [32]. At the vascular level, urea has been shown to alter tight junction proteins in endothelial cells, thereby compromising BBB integrity. Indoxyl sulfate may further activate aryl hydrocarbon receptor signaling in endothelial cells, promoting endothelial activation, inflammatory signaling, adhesion molecule expression, and BBB disruption [30]. Other uremic toxins, including phosphate, p-cresyl sulfate, homocysteine, and oxalic acid, may aggravate endothelial dysfunction by increasing oxidative stress, reducing nitric oxide (NO) bioavailability, upregulating intercellular adhesion molecule (ICAM)-1 and vascular cell adhesion molecule (VCAM)-1 expression, and promoting leukocyte adhesion and transmigration into the CNS [32]. These effects may be reinforced by mitochondrial dysfunction, including excessive mitochondrial reactive oxygen species generation [33,34]. A self-perpetuating cycle may therefore develop in which impaired mitochondrial homeostasis amplifies oxidative stress and inflammatory signaling, further reducing nitric oxide bioavailability, increasing endothelial permeability, and promoting vascular injury [35,36]. In the cerebral circulation, these interrelated abnormalities may disrupt BBB, damage the microvasculature, impair cerebral perfusion, and facilitate neuroinflammation, thereby linking uremic toxicity to cerebrovascular injury and CI [32,37,38,39].

In parallel, uremic toxins may directly affect brain-resident cells, particularly glial cells and neurons. Adesso et al. (2017) showed that indoxyl sulfate activates astrocytes and mixed glial cultures, inducing a pro-inflammatory and pro-oxidative phenotype [40]. This response was characterized by increased expression of inducible NO synthase (iNOS) and cyclooxygenase (COX)-2, enhanced release of tumor necrosis factor (TNF)-α and interleukin (IL)-6, elevated nitrotyrosine formation and reactive oxygen species (ROS) production, and reduced antioxidant defenses, including superoxide dismutase (SOD) [40]. Evidence from a 5/6 nephrectomy model of CKD in mice further suggests that indoxyl sulfate accumulation may promote hippocampal inflammation and NOD-, LRR-, and pyrin domain-containing protein (NLRP)3 inflammasome activation in astrocytes and microglia, ultimately contributing to impaired learning and memory [41]. Moreover, quinolinic acid may induce astrogliosis and stimulate astrocytic production of C-C motif chemokine ligand (CCL)2, CCL5, and IL-8, thereby amplifying local inflammatory cell recruitment [42]. In microglia, kynurenine pathway metabolites, including quinolinic acid, may promote a pro-inflammatory phenotype. Moreover, guanidino compounds can directly affect neurons by inhibiting gamma-aminobutyric acid (GABA)-A signaling and overstimulating glutamate N-methyl-d-aspartate (NMDA) receptors, leading to increased Ca^2+^ influx and excitotoxicity. β2-microglobulin may additionally impair neurogenesis, whereas toxin-induced oxidative stress can promote neuronal apoptosis and cell death [32]. Collectively, these glial and neuronal effects may create a neurotoxic brain microenvironment that contributes to CI in CKD, independently of, but also in interaction with, vascular and BBB injury. Importantly, uremic toxicity and inflammation are not separate processes but mutually reinforcing mechanisms. Uremic toxins can promote oxidative stress, endothelial activation, BBB dysfunction, and glial responses, thereby facilitating the transition from systemic inflammation to neuroinflammation [31].

### 3.3. From Systemic Inflammation to Neuroinflammation

Chronic low-grade systemic inflammation is increasingly recognized as a hallmark of CKD [43]. Under physiological conditions, the kidneys contribute to immune homeostasis by clearing circulating cytokines and bacterial antigens, such as lipopolysaccharides (LPS), thereby limiting systemic inflammation and immune cell activation driven by pro-inflammatory cytokines and pathogen-associated molecular patterns (PAMPs) [44]. CKD is characterized by elevated levels of multiple inflammatory biomarkers, particularly IL-6, TNF-α, and IL-1, which contribute to systemic inflammation, renal injury, and disease progression [45]. Consistent with this, data from the Chronic Renal Insufficiency Cohort study, involving nearly 4000 individuals, revealed that inflammation increases with CKD severity, as lower eGFR and higher urine albumin-to-creatinine ratio (UACR) were independently associated with higher levels of inflammatory biomarkers, particularly IL-6, TNF-α, high-sensitivity C-reactive protein (hs-CRP), and fibrinogen [46]. In addition, it seems that this state may represent a key mechanism linking peripheral kidney dysfunction with CNS injury [30]. As kidney function declines, persistent immune activation, accumulation of pro-inflammatory mediators, oxidative stress, endothelial dysfunction, and BBB impairment may allow peripheral inflammatory signals to affect the CNS [21]. Of note, IL-6 levels have been associated with CI, including executive dysfunction, as well as structural brain changes such as atrophy and concomitant ventricular enlargement [31,47].

Noteworthy, beyond impaired renal clearance of circulating cytokines, the gut–kidney–brain axis has emerged as an important contributor to neuroinflammation in the course of CKD [48,49,50]. Recent two-step Mendelian randomization study conducted by Zhou et al. (2025) identified the gut microbiota as a significant mediator linking CKD with CI, with specific taxa, including Methanobacteriaceae family and the Eubacterium fissicatena group, partially mediating the effects of renal dysfunction on cognitive performance [48]. A seminal study by Vaziri and colleagues (2012) demonstrated that uremia induced by 5/6 nephrectomy in Sprague-Dawley rats caused marked depletion of key colonic epithelial tight-junction proteins, thereby compromising intestinal barrier integrity [51]. Therefore, it facilitates the systemic translocation of LPS and other microbial-derived metabolites that amplify chronic inflammation [50,52]. These circulating inflammatory mediators, including LPS and cytokines, can compromise BBB integrity and enhance microglial activation, linking peripheral immune dysregulation with neuroinflammation and CI [53]. Collectively, incorporation of the gut–kidney–brain axis may provide a more comprehensive mechanistic framework connecting CKD-associated systemic inflammation with the CNS injury and CI [52]. Finally, the study by Shao et al. (2025) suggests that modulation of the gut microbiota may represent a potential therapeutic strategy for CKD-associated CI, as resveratrol improved cognitive performance in a doxorubicin-induced rat model of CKD while reducing circulating LPS and IL-6 levels and affecting microbial balance [54].

### 3.4. Glymphatic Dysfunction and Impaired Brain Waste Clearance

The glymphatic system, also known as the paravascular system, is a relatively new concept of astroglia-dependent clearance pathway through which cerebrospinal fluid (CSF) enters periarterial spaces, exchanges with interstitial fluid, and facilitates the removal of solutes, including amyloid-β and other metabolic waste products [22,55]. Hence, its role has been extensively studied in the pathophysiology of Alzheimer’s disease (AD) and other neurodegenerative conditions [56,57]. Aquaporin (AQP)4 channels localized on astrocytic end-feet are central to glymphatic fluid movement, making astrocytes polarization and neurovascular-unit integrity crucial for efficient brain clearance [56,58]. Given that sleep is a major physiological driver of glymphatic clearance [59], and that sleep disruption remains common in individuals with CKD, this phenomenon may therefore amplify toxin retention, neuroinflammation, and impaired proteostasis in the brain [22].

## 4. Early-to-Moderate CKD (G1–G3)

Since most of the studies investigating CI in CKD rely primarily on eGFR-based staging, we structured the following analysis around GFR categories [21]. Accordingly, stages G1–G3 are discussed as an early-to-moderate CKD, during which kidney dysfunction may already be accompanied by systemic vascular, metabolic, and inflammatory alterations relevant to brain health.

### 4.1. Clinical Landscape: Prevalence and Cognitive Profile

CI is observed even at the earliest stages of CKD, although this group is underrepresented in screening studies [22]. According to a systematic review conducted by Brodski and co-workers in 2018, only three studies analyzed patients with G1–G2 CKD [16]. In the context of early kidney dysfunction, evidence from a British population-based birth cohort suggests that cognitive changes may emerge even when kidney function is relatively preserved. Among adults aged 60–64 years, lower cystatin C-based eGFR, with a median value of 96.8 mL/min/1.73 m^2^, was associated with poorer verbal memory and slower simple and choice reaction times, supporting the concept that brain–kidney interactions may begin before clinically overt CKD develops [60].

Evidence for CI in early-to-moderate CKD is most consistent in the moderate G3 stage. Hailpern et al. (2007) used the National Health and Nutrition Examination Survey (NHANES) III data, and showed that adults aged 20–59 years with moderate CKD, defined as eGFR 30–59 mL/min/1.73 m^2^, had significantly poorer performance in visual attention and learning [61]. This association was further supported by Tsai et al. (2010), who examined a population-based cohort of middle-aged women and found that moderate CKD, corresponding to G3 stage, was associated with reduced cognitive performance despite the absence of advanced kidney failure [62]. Compared with matched controls with eGFR > 60 mL/min/1.73 m^2^ (corresponding to stage G2 or better), women with moderate CKD showed poorer delayed recall and backward digit span performance, indicating deficits in memory, attention, and executive domains.

The pattern of reduced learning/concentration and visual attention, together with lower working memory and executive performance, particularly backward digit span, is suggestive of a fronto-subcortical cognitive profile consistent with slowed processing and executive dysfunction commonly reported in cerebral SVD and vascular CI [63,64].

Additionally, in children and adolescents, mild-to-moderate CKD is associated with measurable neurocognitive vulnerability. Although the mean cognitive performance in the Chronic Kidney Disease in Children cohort remained within the normal range, a substantial proportion of participants showed deficits in intelligence quotient (IQ), academic achievement, attention, or executive function, particularly in the presence of a lower iohexol-based GFR or elevated proteinuria [65]. Interestingly, not all studies have demonstrated a linear worsening of cognitive performance with declining eGFR. In an NHANES 2011–2014 analysis of adults aged ≥60 years, CKD stages G1–G2, defined by preserved eGFR with albuminuria, were associated with impaired immediate memory and poorer digit symbol substitution test performance, whereas CKD stages G3–G5 were not significantly associated with CI after multivariable adjustment [66].

### 4.2. Pathophysiological Mechanisms

In early CKD, CI is likely driven predominantly by vascular and endothelial mechanisms rather than advanced uremic toxicity [30]. Although measurable changes in selected uremic retention solutes may occur already in early CKD, clinically meaningful accumulation of uremic toxins is generally more evident with CKD progression, particularly in G3b–G5 stages [67].

Albuminuria, even in the presence of a relatively preserved eGFR, may reflect systemic endothelial and microvascular dysfunction. Accordingly, it may represent one of the earliest kidney-related markers linking CKD and CI. In an 11-year Finnish population-based follow-up study, micro- and macroalbuminuria were associated with poorer word-list learning, slower reaction times, and greater decline in word-list learning after multivariable adjustment. Notably, even UACR values below the usual threshold for microalbuminuria were associated with poorer verbal fluency [68]. Similar conclusions were drawn from the Prevention of Renal and Vascular End-Stage Disease (PREVEND) study, a community-based cohort of adults aged 35–82 years, in which albuminuria, but not eGFR, was associated with poorer cognitive performance. Notably, this association was evident mainly in the youngest age tertile, suggesting that albuminuria may be a particularly sensitive marker of early microvascular brain vulnerability before age- and comorbidity-related mechanisms become dominant. Moreover, increased albuminuria before cognitive assessment was associated with lower Ruff Figural Fluency Test scores, further supporting the concept that albuminuria may reflect a dynamic systemic endothelial phenotype relevant to cognitive decline [69].

These findings suggest that low-grade albuminuria may capture early microvascular brain vulnerability before advanced CKD-related uremic toxicity becomes the dominant factor. Although uremic toxicity is expected to become more prominent with CKD progression, some evidence suggests that selected toxins may already be relevant in early-to-moderate disease [32,67]. Yeh et al. (2016) showed that serum indoxyl sulfate levels increased progressively across G3–G5 CKD stages and were independently associated with poorer executive function, but this association was statistically significant only in stage G3 of CKD and not in stages G4–G5 [70]. This finding suggests that, in early CKD, indoxyl sulfate may contribute to CI mainly through vascular toxicity, endothelial oxidative stress, or direct neurotoxic effects, whereas in more advanced CKD, its impact may be obscured by other coexisting mechanisms [70].

Another mechanism that may be relevant already in early CKD is impaired neurovascular coupling, defined as the dynamic relationship between neuronal activity and local cerebral blood flow (CBF) responses. Importantly, neurovascular coupling has been shown to be impaired even in early CKD stages, including G1–G3a, and to worsen further in more advanced CKD not requiring KRT, corresponding to stages G3b–G5. Greater impairment of this response is associated with more severe CI, suggesting that altered cerebrovascular reactivity may represent an early functional marker of kidney–brain axis disruption, preceding or accompanying overt structural brain injuries [71]. Finally, in this mechanistic framework, glymphatic dysfunction may represent an additional mechanism that affects cognition in patients with CKD. Since glymphatic clearance depends on intact perivascular pathways, vascular pulsatility, BBB integrity, and astrocytic water transport [72], early endothelial injury, albuminuria-related microvascular dysfunction, and impaired neurovascular coupling may already compromise brain waste clearance before overt uremic neurotoxicity becomes dominant. This mechanism appears particularly plausible given that CSF pulsatility and glymphatic clearance are closely linked to circadian rhythms [72], and that patients with CKD frequently experience sleep disturbances that may further impair their glymphatic function [22,73,74].

Consistent with this concept, Xu et al. (2023) examined 77 conservatively treated, CKD patients not requiring KRT and 50 age-matched healthy controls using magnetic resonance imaging (MRI)-visible enlarged perivascular spaces and ventricular area ratios as indirect markers of glymphatic dysfunction [75]. Individuals with CKD and CI had a greater burden of enlarged perivascular spaces, particularly in the frontal cortex and basal ganglia, as well as larger lateral and fourth ventricle area ratios, and these abnormalities correlated negatively with Mini-Mental State Examination (MMSE) and Montreal Cognitive Assessment (MoCA) scores. Although the detailed CKD stage distribution was not reported in this study, renal function differed across the cognitive subgroups. Patients with CI had a lower mean eGFR than cognitively intact CKD patients (57.5 ± 31.8 vs. 65.4 ± 38.0 mL/min/1.73 m^2^), and within the CI group, those classified as having dementia showed substantially lower eGFR than those with mild CI (44.4 ± 23.7 vs. 69.3 ± 34.0 mL/min/1.73 m^2^). This pattern suggests that glymphatic abnormalities may become more clinically relevant as renal dysfunction progresses from relatively preserved or mildly reduced kidney function toward moderate CKD [75]. However, because the study did not provide a formal G-stage distribution, these findings should be interpreted as supporting an eGFR-related gradient, rather than a stage-specific threshold effect.

## 5. Advanced CKD (G4–G5 Not Requiring KRT)

### 5.1. Clinical Landscape: Escalation of Cognitive Burden

In advanced CKD not requiring KRT, corresponding to stages G4–G5, CI appears to become more frequent, clinically evident, and increasingly multidomain. In a systematic review and meta-analysis by Etgen et al. (2012), CKD was associated with a significantly higher risk of CI in both cross-sectional (OR: 1.65; 95% Confidence Interval: 1.32–2.05), and longitudinal studies (OR: 1.39; 95% Confidence Interval: 1.15–1.68), supporting CKD as an independent risk factor for CI [15]. Importantly, sensitivity analyses suggested that the risk was more pronounced in moderate-to-severe CKD, especially when eGFR declined below 45 mL/min/1.73 m^2^ compared to G3a stage [15]. This stage-dependent pattern was further supported by Brodski et al. (2019), who systematically reviewed non-elderly (<65 years of age) CKD cohorts and found that cognitive functions deteriorated progressively from stage G1 to stage G5, with early CKD mainly affecting processing speed, attention, response speed, and short-term memory, whereas stage G5 disease was associated with broader impairment involving executive functions, immediate and delayed memory, visuospatial function, and global cognition [16]. In a cross-sectional study by Egbi et al. (2015), involving 190 individuals with CKD and 100 healthy controls, a clear increase in the prevalence of CI across CKD stages, from 24.0% in stage G3 to 41.6% in stage G4 and 46.2% in stage G5, was reported, illustrating the clinical escalation of cognitive burden with advancing kidney dysfunction [76]. Also, Levassort and colleagues (2024) reported findings from a large longitudinal cohort study of more than 3000 individuals with G3–G4 stages of CKD and intact cognitive function at baseline, demonstrating significant declines in orientation, language, and praxis over a 5-year follow-up period [77].

Complementing these findings, a meta-analysis conducted by Berger et al. (2016), involving only individuals with CKD not requiring KRT, showed that individuals with eGFR <60 mL/min/1.73 m^2^ performed worse than controls across several cognitive domains [78]. Stratified analyses suggested that language deficits became more pronounced at lower eGFR thresholds, whereas executive dysfunction was most apparent when eGFR declined below 30 mL/min/1.73 m^2^, corresponding to advanced pre-dialysis stage G4–G5 CKD. Together, these findings indicate that the progression from moderate to advanced CKD is associated with broader cognitive vulnerability, although the pattern of impairment varies across the cognitive domains [78].

Finally, Liu et al. (2020) reported that CI becomes more pronounced with advancing CKD not yet requiring KRT, with stage G4 CKD patients demonstrating significantly lower MoCA, MMSE, and digit symbol test scores than healthy controls [79]. These cognitive deficits, particularly reduced processing speed, were associated with white matter microstructural abnormalities, including decreased fractional anisotropy and increased mean diffusivity in the corpus callosum and major association tracts [79].

Although stage G5 of CKD was not examined, the findings support a progressive kidney–brain relationship in which worsening renal dysfunction is accompanied by increasing white matter injury and CI [79]. Together with evidence linking reduced renal function to WMHs, impaired white matter integrity, and executive dysfunction, these findings are consistent with a predominantly vascular and frontal–subcortical model of CKD-related CI. Nonetheless, concomitant associations with cortical atrophy and reduced hippocampal volume suggest that more advanced or prolonged renal dysfunction may also involve broader brain vulnerability beyond SVD alone [80]. Thus, the available evidence supports an expansion from selective vascular-subcortical deficits to multidomain impairment.

### 5.2. Pathophysiological Mechanisms

The clinical escalation of CI in advanced CKD not requiring KRT suggests that declining kidney function is accompanied not only by a greater cognitive burden but also by the increasing convergence of multiple kidney-related pathophysiological insults. This interpretation is consistent with the kidney–brain axis framework, in which vascular injury, endothelial dysfunction, inflammation, oxidative stress, BBB disruption, uremic toxin accumulation, and brain structural changes may jointly contribute to CKD-related cognitive decline. While microvascular injury and impaired cerebrovascular regulation may be particularly relevant in the earlier stages of CKD, more advanced renal dysfunction is likely to amplify neurotoxic and inflammatory pathways, including the accumulation of uremic solutes such as indoxyl sulfate [21]. These toxins may promote endothelial activation, oxidative stress, BBB disruption, inflammatory signaling, and potentially direct neuronal or glial injury, thereby linking progressive renal clearance failure to cerebrovascular and neurocellular damage [29,81]. Experimental evidence supports the potential contribution of uremic toxin-mediated hippocampal injury to CKD-related CI. For instance, Watanabe et al. (2021) showed that several uremic toxins, including indoxyl sulfate, indole, 3-indoleacetate, and methylglyoxal, reduced viability and glutathione levels in a mouse hippocampal neuronal cell line (HT-22), whereas rats with adenine-induced CKD exhibited oxidative stress and pyknosis in the hippocampus [82]. Furthermore, in an ex vivo electrophysiological model using rat hippocampal CA1 slices, Natale et al. (2021) showed that guanidine and serum from dialysis patients enhanced glutamatergic transmission, including NMDA- and α-amino-3-hydroxy-5-methyl-4-isoxazolepropionic acid (AMPA)-mediated currents, suggesting a potential excitotoxic mechanism [83].

Such excessive glutamatergic activity may increase intracellular calcium influx and promote neuronal dysfunction or death [64,84], thereby providing a plausible link between uremic toxin accumulation, hippocampal neurodegeneration, and broader CI profile in moderate-to-severe CKD. In addition, in G4–G5 stages of CKD, glymphatic dysfunction may be incorporated into a broader mechanistic framework in which vascular endothelial injury, impaired neurovascular coupling, chronic inflammation, oxidative stress, anemia, and uremic toxin accumulation jointly contribute to CI [22]. This concept is supported by Ko et al. (2024), who examined 56 patients with CKD and 38 healthy controls, with the CKD cohort predominantly composed of stage G5 patients (*n* = 39; 69.6%) [85]. Using diffusion tensor imaging along the perivascular space, diffusion tensor image analysis along the perivascular space (DTI-ALPS), they showed that patients with CKD had lower DTI-ALPS indices than controls, with the greatest reduction observed in those with CI. Thus, glymphatic dysfunction may amplify CKD-related CI, bridging advanced uremic, inflammatory, and vascular mechanisms with multidomain cognitive decline.

At the same time, anemia becomes increasingly relevant in advanced CKD, as its prevalence enhances with the progression of the disease [86,87], and may contribute to CI by reducing cerebral oxygen delivery and compromising the brain’s metabolic reserve [88]. In a cross-sectional analysis of nearly 2300 adults from NHANES 2011–2014, Blasco-Colmenares et al. (2024) found that among participants with CKD G3–G5 not requiring KRT, anemia was associated with impairment in semantic verbal fluency, processing speed, sustained attention, and working memory, suggesting it may predominantly affect executive-attentional aspects of cognition [89]. On the other hand, Kurella Tamura et al. (2016) did not reveal an independent association between anemia and changes in cognitive function among participants in the Chronic Renal Insufficiency Cohort (CRIC) study [90]. Thus, CI in advanced CKD likely results from the cumulative interaction of vascular injury, uremic toxicity, anemia-related factors, inflammation, oxidative stress, and impaired BBB integrity. Noteworthy, contemporary anemia therapies may influence these pathways through distinct mechanisms [88,91]. Erythropoiesis-stimulating agents act through direct activation of the erythropoietin receptor and may additionally confer neurotrophic benefits [88]. A recent systematic review by Barbieri et al. (2024) suggested that recombinant human erythropoietin may improve CKD-associated CI [92]. Nevertheless, treatment strategies aimed at achieving higher hemoglobin concentrations have been linked to an increased risk of hypertension and stroke among individuals with CKD [93]. Hypoxia-inducible factor (HIF) prolyl hydroxylase inhibitors represent an alternative approach by stabilizing HIF and promoting a broader adaptive response to hypoxia, including stimulation of endogenous erythropoietin production, enhancement of iron availability, and metabolic adaptation [88,94]. Although these mechanisms may have neuroprotective and cerebrovascular benefits, the supporting evidence remains largely preclinical [94,95]. Iron supplementation may also contribute to cognitive health by correcting iron deficiency and supporting iron-dependent neuronal metabolism, but CKD-specific clinical evidence is still scarce [88].

## 6. Dialysis-Dependent CKD

### 6.1. Clinical Landscape: Cognitive Instability in Dialysis

In dialysis-dependent CKD, CI becomes highly prevalent and clinically unstable, reflecting the combined effects of kidney failure itself, comorbidity burden, and dialysis-related physiological stress. Among CKD populations, the burden of CI appears to be highest in patients undergoing HD, with prevalence estimates exceeding 50% [96,97]. According to a meta-analysis by Zhang et al. (2024) involving over 25,000 individuals with CKD, CI was present in 53% and 39% of HD and PD patients, respectively, compared to 32% and 26% amongst patients with CKD not requiring KRT and KTx recipients [18]. The authors emphasized age, comorbid diabetes mellitus, and hypertension as the main factors that increase the risk of CI [18]. Interestingly, according to a meta-analysis by O’Lone and co-workers (2016), which analyzed 42 studies involving more than 3500 participants, individuals treated with HD performed better than CKD patients not requiring KRT in the attention and memory domains [98]. It might reflect that some cognitive deficits related to severe uremia may be partially reversible after dialysis initiation [98].

In contrast, Kurella Tamura et al. (2017) assessed cognitive trajectories among 212 participants from the CRIC study in a longitudinal manner, and revealed that dialysis initiation was linked to a decline in executive function without improvement in other aspects of cognitive domains [99,100]. This is in line with other research, including our previously published study, which revealed that executive dysfunction might occur before global cognitive deterioration in the dialysis population [101,102]. Furthermore, studies assessing cognitive function in patients with CKD have consistently demonstrated impaired verbal fluency, suggesting dysfunction of executive and fronto-subcortical networks [9,97,103]. In a cross-sectional study involving patients receiving PD or HD and healthy controls, Sánchez-Fernández et al. (2024) found that the PD group outperformed the HD group across several executive domains, including verbal fluency, working memory, cognitive flexibility, planning, and decision-making [104]. However, cognitive performance in the PD group remained inferior to that of healthy controls, suggesting that dialysis modality may influence the severity of CI rather than its occurrence [104]. However, in a large cohort of HD patients, CI was considerably more frequent on tests of memory and executive function than on the Controlled Oral Word Association Test (COWAT), a measure of phonemic verbal fluency, emphasizing that executive dysfunction may extend beyond deficits captured by verbal fluency tasks alone [105].

Nevertheless, patients treated with HD had worse scores than the general population across several cognitive domains, exceeding executive dysfunction, with the most pronounced deficit observed in orientation and attention, followed by impairments in memory [98]. Interestingly, individuals who initiated HD within one year, revealed predominantly the impairment of verbal skills (55%), and reasoning (43%), while only less than 20% had deficits in short-term memory [106]. It is in line with studies suggesting that dialysis vintage is one of the most significant contributors to CI in this population [107,108]. For example, Gangasani et al. (2025) reported a high prevalence of 76.8% in the HD cohort, with patients who had a dialysis vintage of less than 24 months exhibiting significantly higher scores in Addenbrooke’s Cognitive Examination-III than those with a dialysis vintage greater than 72 months (84 ± 6 vs. 61 ± 7; *p* = 0.001) [107]. Although numerous strategies have been proposed to improve cognitive function in the HD population, Abdelwahab et al. (2022) found that intradialytic exercise and cooled dialysate, two potentially beneficial interventions, had no significant effect on the mean MoCA scores [109].

CI is also common among patients treated with PD, however the clinical context differs because PD is a home-based therapy that requires intact memory, executive function, and procedural skills [110,111]. An initial systematic review conducted by Shea et al. (2019), involving over 1700 patients treated with PD, reported a pooled CI prevalence of 28.7% (95% Confidence Interval: 15.9–46%) [112]. Importantly, they showed that CI was linked to an enhanced risk of hospitalization, mostly due to PD-related peritonitis [112]. A more recent meta-analysis has placed this prevalence even higher, with Aiumtrakul and colleagues (2024) reporting a prevalence of 47.7% (95% Confidence Interval: 35.8–59.9%) [113]. A cross-sectional study by Shin et al. (2020) demonstrated that high relative overhydration, a proxy for inadequate dialysis, was associated with executive dysfunction in patients receiving PD [114]. Similarly, Li and co-workers (2020) reported that greater middle molecule clearance was independently associated with better global cognitive performance and executive function in individuals treated with PD [115]. Collectively, these findings underscore the importance of adequate dialysis in preserving cognitive function.

Notably, individuals on PD showed a trend toward a lower risk of CI than those undergoing HD (OR 0.64; 95% Confidence Interval: 0.39–1.03; *p* = 0.068) [113]. Research has consistently suggested that PD-treated individuals score higher on cognitive screening batteries, such as the MMSE and MoCA, and have a lower risk of dementia than those treated with HD [116]. The most up-to-date meta-analysis, conducted by Malik et al. (2026), provided one of the most comprehensive assessments of the effect of dialysis modality on cognitive outcomes, including 26 studies and more than 300,000 patients [117]. Overall, the findings favored PD over HD, particularly with respect to executive function, memory, processing speed, and long-term cognitive outcomes, including dementia incidence [117]. Concluding, dialysis-dependent CKD should be viewed as a phase of cognitive vulnerability and fluctuation, in which baseline multidomain impairment is compounded by modality-specific stressors and clinically relevant changes in attention, memory, executive function, and capacity for self-management of treatment.

### 6.2. Pathophysiological Mechanisms

In dialysis-dependent CKD, CI is driven not only by persistent uremic toxicity and vascular comorbidities but also by dialysis-specific physiological stressors. Notably, these drivers differ substantially between individuals treated with PD and HD [118]. HD exposes the CNS to recurrent episodes of circulatory stress, rapid solute removal, osmotic shifts, inflammatory activation, and fluctuations in cerebral oxygen delivery, all of which may contribute to cognitive instability during and between dialysis sessions. Patients undergoing HD are particularly vulnerable because advanced CKD is frequently accompanied by vascular stiffness, impaired cerebrovascular reserve, endothelial dysfunction, anemia, and cerebral SVD, which reduce the brain’s capacity to tolerate acute hemodynamic and metabolic stress [118,119,120,121]. Of note, anemia affects up to 90% of patients receiving maintenance HD [122], while a recent study by Huang et al. (2025) emphasized that lower hemoglobin levels were independently associated with CI amongst these individuals [123].

In contrast, PD may be characterized by gentler hemodynamic shifts and typically avoids routine systemic anticoagulation [13]. Nevertheless, glucose-based peritoneal dialysate may contribute to metabolic stress, including intermittent hyperglycemia and increased exposure to advanced glycation end products (AGEs) [118].

#### 6.2.1. Hemodynamic Injury

Hemodynamic injury is one of the most important dialysis-specific mechanisms linking HD to cognitive dysfunction. During HD sessions, rapid ultrafiltration, changes in plasma volume, and intradialytic blood pressure instability may reduce cerebral perfusion, particularly in patients with impaired autoregulation and pre-existing cerebrovascular diseases [13,118,121]. Polinder-Bos et al. (2018) assessed the effect of HD on CBF using positron emission tomography-computed tomography (PET-CT) in a small cohort of 12 patients [124]. They demonstrated a decrease in global CBF, as well as regional reductions affecting the frontal, parietal, temporal, and occipital lobes, cerebellum, and thalamus [124]. Given that chronic cerebral hypoperfusion (CCH) constitutes one of the major drivers of vascular CI [64,125], recurrent reductions in cerebral oxygenation may preferentially compromise watershed regions, white matter tracts, and frontal–subcortical circuits, thereby affecting cognitive domains, such as attention, processing speed, and executive function [121]. This seems particularly clinically relevant because CBF has been shown to decline acutely during HD, and intradialytic hypotension has been linked to cerebral ischemia [120,126].

MacEwen and colleagues (2017) indicated that intradialytic cerebral ischemia, defined as a 15% decrease from baseline cerebral oxygen saturation, was associated with changes in executive cognitive function, measured using the Trail Making Test [126]. According to the Vascular Impairment of Cognition Classification Consensus Study (VICCCS)-2 and Vascular Behavioral and Cognitive Disorders (VasCog)-2 criteria, vascular CI typically affects executive function, attention, memory, language, visuospatial function, and processing speed [127,128]. Therefore, hemodynamic injury may provide a plausible explanation for the predominance of impairment in these domains among patients undergoing HD [120]. Finally, hemodynamic mechanisms may also explain the fluctuations in cognitive performance observed during HD and off-dialysis periods [119]. The seminal study by Murray et al. (2007) showed that cognitive performance was highest during the interdialytic period, 24 to 30 h after dialysis, followed by 1 h before HD, and was lowest 45 to 90 min after the start of the dialysis session [129]. Similarly, Findlay et al. (2019) reported that individuals on HD performed worse on assessments of executive function during dialysis and on measures of attention and task-switching compared with off-dialysis assessments [130]. This hemodynamic interpretation is further supported by the observed intradialytic decline in cerebral mean flow velocity (MFV) on transcranial Doppler ultrasound, which correlates with ultrafiltration volume and deterioration in global cognition, executive function, and verbal fluency. MRI findings also highlight cumulative cerebrovascular injury, as patients who remained on HD during follow-up demonstrated progression of WMHs burden and lobar atrophy in the frontal, parietal, and temporal regions. Importantly, at 12 months, greater MFV decline was associated with worse global and executive function among patients continuing HD, whereas transplanted patients showed no WMHs progression and demonstrated improvement in memory. These findings suggest that HD-related cerebral hypoperfusion may contribute not only to transient intradialytic cognitive fluctuations but also to longer-term structural brain injury [130].

#### 6.2.2. Osmotic and Electrolyte Shifts

Historically, dialysis disequilibrium syndrome (DDS) has been described as a particularly severe neurological disturbance associated with a rapid reduction in plasma urea levels relative to brain urea levels. This creates an osmotic gradient that drives water into the brain and causes acute cerebral oedema. [131,132]. Although overt DDS is now uncommon, milder osmotic shifts may still contribute to transient cognitive symptoms during or after HD due to cerebral edema or brain tissue shrinkage [8]. Sodium shifts may be particularly relevant because dialysate sodium prescriptions can influence plasma sodium trajectories, interdialytic weight gain, hemodynamics, and osmotic stability [118]. Controlled sodium algorithms have been shown to minimize plasma sodium shifts during HD, supporting the concept that dialysis prescriptions can modulate osmotic stress [133].

Additionally, PD leads to continuous exposure to glucose-based dialysate, which may eventually cause hyperglycemia and dyslipidemia [118]. Constant absorption of glucose and glucose degradation products (GDP) promotes the formation of AGEs [134,135,136], which are associated with microvascular injury and have also been linked to CI [137,138]. Evidence suggests that the adverse metabolic consequences of conventional glucose-based dialysate may be partially mitigated by the use of biocompatible neutral-pH, low-GDP solutions and glucose-sparing strategies incorporating icodextrin [139,140]. A randomized trial by Schmitt et al. (2007) demonstrated that switching from conventional high-GDP to low-GDP dialysate significantly reduced circulating AGEs [141]. Similarly, le Poole et al. (2012) reported that glucose-sparing PD regimens attenuated increases in selected carbonyl stress markers, including 3-deoxyglucosone and Nε-carboxyethyl-lysine, although reductions were not observed consistently across all AGE-related compounds [142]. Furthermore, Li and colleagues (2013) demonstrated that glucose-sparing prescriptions incorporating icodextrin improved glycemic control and reduced triglyceride, very-low-density lipoprotein, and apolipoprotein B levels in diabetic patients receiving PD [143]. Collectively, these findings suggest that modern biocompatible dialysate formulations may partially mitigate glucose-induced metabolic and microvascular stress. However, despite this strong biological rationale, no clinical studies have yet demonstrated that these interventions translate into improved cognitive performance or reduced long-term risk of CI.

## 7. Post-Transplantation

### 7.1. Clinical Landscape: Partial Recovery and Residual Deficits

KTx is increasingly considered the gold standard treatment for individuals with kidney failure [144,145]. Hence, it represents a completely different stage in the kidney–brain trajectory, in which CI may partially recover after restoration of kidney function, but often does not fully normalize [146,147]. The prevalence of CI in this population varies substantially across studies, ranging from 15.6% to as high as 58.0% [148,149,150,151]. Importantly, cognitive functions might at least partially improve following KTx [152,153]. In a meta-analysis conducted by Joshee et al. in 2018, which included 10 studies, KTx recipients showed improvements in general cognitive status, information and motor speed, spatial reasoning, verbal memory, and visual memory relative to their pre-KTx performance [152]. However, they continued to perform worse than the general population. More recent longitudinal data further support the domain-specific pattern of recovery. Gupta et al. (2024) assessed cognitive function before KTx, at 3 months post-KTx, and at 1 year post-KTx using a comprehensive neuropsychological battery [153]. KTx recipients presented normalization of episodic and verbal declarative memory, whereas persistent deficits or lack of measurable improvement were observed in global cognition, working memory, attention, and selected executive domains [153].

Taken together, these findings suggest that CI in CKD might be partly reversible, although recovery is not uniform across all cognitive domains. Preferential improvement in memory and processing-related domains may reflect the attenuation of potentially reversible metabolic, inflammatory, and uremic factors after KTx. In contrast, persistent impairment in executive function, attention, language, and verbal fluency may reflect a more durable brain injury, including cerebrovascular disease, WMHs, lacunar infarcts, brain atrophy, or SVD. Residual impairment may also be sustained by post-KTx factors, including hypertension, diabetes, dyslipidemia, metabolic syndrome, hyperuricemia, lower graft function, and immunosuppressive therapy, particularly with tacrolimus [147]. Therefore, KTx should not be conceptualized as complete neurocognitive recovery but rather as a phase of partial cognitive restoration with persistent vulnerability in selected domains, which could continue to affect adherence, medication management, and long-term clinical outcomes.

### 7.2. Pathophysiological Mechanisms

On the one hand, improved kidney function after KTx reduces the retention of uremic solutes, stabilizes internal homeostasis, improves anemia and acid-base balance, and eliminates dialysis-related hemodynamic stress. However, KTx recipients remain exposed to vascular risk, chronic inflammation, infections, perioperative and metabolic complications, and neurotoxic immunosuppressive agents.

#### 7.2.1. Reversal of Uremic Neurotoxicity

CI observed after KTx supports the concept that a component of CKD-related CI is functionally reversible, with the rapid reduction in uremic toxin levels representing a potential mechanism for this phenomenon [154]. This explanation is biologically plausible because uremic toxins are known to affect endothelial function, BBB permeability, and glial inflammatory activation [155]. Interestingly, improvement in cerebral perfusion and white matter integrity after transplantation suggests that brain recovery involves not only metabolic detoxification, but also vascular hemodynamic normalization to some extend [156].

#### 7.2.2. Immunosuppressive Neurotoxicity

Of note, CNI, widely used immunosuppressive agents in KTx recipients, especially tacrolimus and cyclosporine, might cause a vast spectrum of neurotoxicity, ranging from tremors and headaches to seizures, encephalopathy, posterior reversible encephalopathy syndrome, and cognitive symptoms [146,157].

Mechanistically, CNI neurotoxicity may involve endothelial dysfunction, vasoconstriction, BBB disruption, mitochondrial stress, and direct neuronal excitability changes [157]. Tacrolimus formulation and exposure may influence CBF and cognitive function in KTx recipients, suggesting that pharmacokinetic variability may be relevant to neurocognitive outcomes, with once-daily extended-release formulations having beneficial effects [158]. Interestingly, Martínez-Sanchis and colleagues (2011) showed that KTx recipients receiving sirolimus or tacrolimus received lower scores in attention and working memory than individuals treated with cyclosporine and the control group [159].

Finally, glucocorticosteroids may further contribute to mood disturbances, sleep disruption, and metabolic risk factors that indirectly impair cognition [148,157]. Our previously published study revealed that KTx recipients treated with higher daily doses of oral prednisone were more likely to develop both depression and anxiety, which are known to be associated with CI as well [148].

## 8. Translational Implications and Future Directions

First of all, there is still a lack of consensus and validation for screening batteries dedicated to individuals with CKD and cognitive decline [17]. Additionally, future research should move from global cognitive screening toward stage- and domain-specific neurocognitive phenotyping that distinguishes vascular-executive, uremic-toxic, inflammatory, dialysis-hemodynamic, and post-KTx medication-related profiles [160]. Multimodal biomarker panels that integrate eGFR, albuminuria, uremic toxin profiles, inflammatory and endothelial biomarkers, neurofilament light chain, MRI, cerebral perfusion imaging, electroencephalography (EEG)-derived measures such as cortical slowing, altered spectral power, functional connectivity, and digital cognitive monitoring may improve the detection of early kidney–brain injury [161].

The optimal control of vascular risk, albuminuria, blood pressure, diabetes, sleep apnea, depression, and inflammatory burden may be the most realistic strategies for cognitive prevention [8]. This approach should also include adequate nutritional support and physical activity programs [8], as a sedentary lifestyle remains one of the key modifiable risk factors for CI, particularly among dialysis patients and KTx recipients [162]. Given that even subclinical CI is associated with frailty, impaired mobility, and reduced quality of life in CKD, interventions promoting physical function may provide benefits beyond cardiovascular risk reduction [163]. Nutritional interventions are also of interest, as a high-protein diet may be associated with memory impairment in individuals with G1-G2 CKD, whereas low protein intake may be associated with depression in advanced CKD [164]. In transplant recipients, long-term longitudinal studies are warranted to clarify which deficits recover after uremia reversal and which are sustained by vascular disease, aging, perioperative insults, or immunosuppressive neurotoxicity [17,147]. Ultimately, the stage-dependent kidney–brain framework proposed in this review may support precision medicine by enabling stage-specific biomarker selection, individualized cognitive monitoring, and mechanism-oriented therapeutic interventions, rather than treating CKD-associated CI as a single clinical entity. Accordingly, early CKD management could focus on mitigating vascular and inflammatory risk factors, advanced CKD management on targeting uremic neurotoxicity, dialysis care on minimizing repetitive cerebral stress, and post-KTx care on balancing the benefits of detoxification against the potential neurotoxic effects of immunosuppressive therapy [21,22,30,118,147].

Nevertheless, several important limitations should be acknowledged. Most available evidence is derived from observational studies, limiting causal inference between molecular alterations and cognitive outcomes. Furthermore, substantial heterogeneity in CKD populations, cognitive assessment tools, and study designs complicates comparisons across studies. Many proposed mechanisms, including glymphatic dysfunction, mitochondrial injury, and BBB disruption, are currently supported predominantly by experimental or indirect evidence, whereas longitudinal human studies integrating molecular biomarkers, neuroimaging, and domain-specific cognitive assessment remain scarce. Consequently, prospective multimodal studies and interventional clinical trials are required to validate the proposed stage-dependent kidney–brain model before it can be translated into routine clinical practice.

## 9. Conclusions

CI is observed across the entire spectrum of CKD, however its pathogenesis appears to differ depending on the disease stage, reflecting the variable contributions of vascular injury, uremic toxicity, oxidative stress, inflammation, dialysis-related hemodynamic stress, and post-KTx medication-related neurotoxicity. As CKD progresses, these mechanisms increasingly overlap and interact, resulting in a multifactorial pathophysiological process rather than clearly separable, stage-specific entities. In early-to-moderate CKD, endothelial dysfunction, albuminuria-associated microvascular injury, and impaired neurovascular coupling predominantly affect frontal–subcortical networks, leading mainly to deficits in attention, executive function, processing speed, and working memory. In advanced CKD, progressive accumulation of uremic toxins, BBB disruption, oxidative stress, systemic and neuroinflammation, anemia, and impaired brain waste clearance broaden the cognitive phenotype to include memory, language, visuospatial function, and global cognition. In dialysis-dependent CKD, these chronic molecular disturbances are compounded by modality-specific mechanisms. HD-related cerebral hypoperfusion, osmotic shifts, and recurrent ischemic stress promote fluctuating executive, attentional, and memory deficits, whereas PD is associated with persistent uremic-inflammatory and metabolic stress, resulting in generally milder but still multidomain CI. Following KTx, reduction in uremic toxicity and restoration of metabolic homeostasis may improve memory and processing-related functions, while persistent vascular injury and immunosuppressive neurotoxicity may sustain deficits in attention and executive function. Thus, the type and severity of CI in CKD are determined by the dominant molecular and physiological disturbances at each disease stage. Therefore, CI in CKD should be understood as a continuum of kidney–brain injury, in which distinct mechanisms may predominate at different stages, but rarely act in isolation.

## Figures and Tables

**Table 1:** Stage-dependent mechanisms, cognitive profiles, and potential intervention targets in CKD-associated cognitive impairment.

CKD Stage	Pathomechanism	Typical Cognitive Profile	Potential Intervention Focus
Early-to-Moderate CKD (G1–G3)	Endothelial injury, albuminuria-related microvascular dysfunction, impaired neurovascular coupling, early glymphatic vulnerability	Usually **subtle** fronto-subcortical deficits: attention, executive function, processing speed, working memory	Vascular risk control, albuminuria reduction, sleep optimization, prevention of early microvascular injury
Advanced CKD (G4–G5 not requiring KRT)	Uremic toxicity, the BBB disruption, oxidative stress, inflammation, anemia, glymphatic dysfunction, white matter injury	**Increasingly frequent multidomain** impairment: executive function, attention, processing speed, memory, language, visuospatial function, global cognition	Reduction in uremic, inflammatory, oxidative, vascular, and anemia-related brain injury
PD-dependent CKD	Persistent uremic-inflammatory burden, metabolic stress from glucose-based dialysate, AGEs, infection risk	CI **common but less frequent than in HD**; multidomain deficits affecting memory, attention, executive function, processing speed	Peritonitis prevention, metabolic optimization, self-management support
HD-dependent CKD	Intradialytic hypoperfusion, BP instability, ultrafiltration stress, osmotic/electrolyte shifts, inflammation, cumulative cerebrovascular injury	**Highest CI burden in all CKD** groups; fluctuating cognition with executive dysfunction, attention deficits, verbal fluency impairment, processing speed and memory deficits	Hemodynamic stabilization, avoidance of intradialytic hypotension, individualized ultrafiltration, osmotic-stress reduction
Post-KTx	Partial uremic reversal, improved homeostasis, persistent SVD, vascular comorbidity, immunosuppressive neurotoxicity	CI prevalence lower than in dialysis; **partial recovery with residual deficits**, memory may improve, attention and executive deficits may persist	Vascular risk control, graft-function preservation, immunosuppression monitoring, mood and sleep management

AGEs: advanced glycation end-products; BBB: blood–brain barrier; BP: blood pressure; CI: cognitive impairment; CKD: chronic kidney disease; G1–G5: glomerular filtration rate categories 1–5; HD: hemodialysis; KRT: kidney replacement therapy; KTx: kidney transplantation; PD: peritoneal dialysis; SVD: small vessel disease.

## Data Availability

All data supporting the findings of this study are available within the paper and can be accessed by DOI from references.

## Abbreviations

The following abbreviations are used in this manuscript:

AD	Alzheimer’s disease
AGEs	advanced glycation end products
AMPA	α-amino-3-hydroxy-5-methyl-4-isoxazolepropionic acid
AQP4	aquaporin 4
BBB	blood–brain barrier
CBF	cerebral blood flow
CCH	chronic cerebral hypoperfusion
CCL	C-C motif chemokine ligand
CGA	Cause/Glomerular filtration rate/Albuminuria
CI	cognitive impairment
CKD	chronic kidney disease
CNI	calcineurin inhibitors
CNS	central nervous system
COX-2	cyclooxygenase-2
CRIC	Chronic Renal Insufficiency Cohort
CSF	cerebrospinal fluid
DDS	dialysis disequilibrium syndrome
DTI-ALPS	diffusion tensor image analysis along the perivascular space
EEG	electroencephalography
eGFR	estimated glomerular filtration rate
GABA-A	gamma-aminobutyric acid A
GDP	glucose degradation products
GFR	glomerular filtration rate
HD	hemodialysis
hs-CRP	high-sensitivity C-reactive protein
ICAM-1	intercellular adhesion molecule-1
IL	interleukin
iNOS	inducible nitric oxide synthase
IQ	intelligence quotient
KDIGO	Kidney Disease: Improving Global Outcomes
KRT	kidney replacement therapy
KTx	kidney transplantation
MFV	mean flow velocity
MMSE	Mini-Mental State Examination
MoCA	Montreal Cognitive Assessment
MRI	magnetic resonance imaging
NHANES	National Health and Nutrition Examination Survey
NLRP3	NOD-, LRR-, and pyrin domain-containing protein 3
NMDA	N-methyl-D-aspartate
NO	nitric oxide
OR	odds ratio
PAMPs	pathogen-associated molecular patterns
PD	peritoneal dialysis
PET-CT	positron emission tomography-computed tomography
PREVEND	Prevention of Renal and Vascular End-Stage Disease
ROS	reactive oxygen species
SOD	superoxide dismutase
SVD	small vessel disease
TNF-α	tumor necrosis factor-α
UACR	urine albumin-to-creatinine ratio
VasCog-2	Vascular Behavioral and Cognitive Disorders-2
VCAM-1	vascular cell adhesion molecule-1
VICCCS-2	Vascular Impairment of Cognition Classification Consensus Study-2
WMHs	white matter hyperintensities
